# The P2Y_12_ Antagonists, 2MeSAMP and Cangrelor, Inhibit Platelet Activation through P2Y_12_/G_i_-Dependent Mechanism

**DOI:** 10.1371/journal.pone.0051037

**Published:** 2012-12-06

**Authors:** Binggang Xiang, Guoying Zhang, Hongmei Ren, Manjula Sunkara, Andrew J. Morris, T. Kent Gartner, Susan S. Smyth, Zhenyu Li

**Affiliations:** 1 Saha Cardiovascular Research Center, College of Medicine, University of Kentucky, Lexington, Kentucky, United States of America; 2 Department of Biology, University of Memphis, Memphis, Tennessee, United States of America; 3 Lexington VA Medical Center, Lexington, Kentucky, United States of America; Chang Gung University, Taiwan

## Abstract

**Background:**

ADP is an important physiological agonist that induces integrin activation and platelet aggregation through its receptors P2Y_1_ (Gα_q_-coupled) and P2Y_12_ (Gα_i_-coupled). P2Y_12_ plays a critical role in platelet activation and thrombosis. Adenosine-based P2Y_12_ antagonists, 2-methylthioadenosine 5′-monophosphate triethylammonium salt hydrate (2MeSAMP) and Cangrelor (AR-C69931MX) have been widely used to demonstrate the role of P2Y_12_ in platelet function. Cangrelor is being evaluated in clinical trials of thrombotic diseases. However, a recent study reported that both 2MeSAMP and Cangrelor raise intra-platelet cAMP levels and inhibit platelet aggregation through a P2Y_12_-independent mechanism.

**Methodology/Principal Findings:**

The present work, using P2Y_12_ deficient mice, sought to clarify previous conflicting reports and to elucidate the mechanisms by which 2MeSAMP and Cangrelor inhibit platelet activation and thrombosis. 2MeSAMP and Cangrelor inhibited aggregation and ATP release of wild-type but not P2Y_12_ deficient platelets. 2MeSAMP and Cangrelor neither raised intracellular cAMP concentrations nor induced phosphorylation of vasodilator-stimulated phosphoprotein (VASP) in washed human or mouse platelets. Furthermore, unlike the activators (PGI_2_ and forskolin) of the cAMP pathway, 2MeSAMP and Cangrelor failed to inhibit Ca^2+^ mobilization, Akt phosphorylation, and Rap1b activation in P2Y_12_ deficient platelets. Importantly, while injection of Cangrelor inhibited thrombus formation in a FeCl_3_-induced thrombosis model in wild-type mice, it failed to affect thrombus formation in P2Y_12_ deficient mice.

**Conclusions:**

These data together demonstrate that 2MeSAMP and Cangrelor inhibit platelet function through the P2Y_12_-dependent mechanism both *in vitro* and *in vivo*.

## Introduction

Platelets play a key role in hemostasis. Platelet activation includes a series of positive feedback loops that rapidly amplify activation signals to enable robust platelet recruitment and stabilization of thrombi at the sites of vascular injury. Two important mechanisms for amplification are the release of granule cargo (mainly ADP) and synthesis of TXA_2_ from cyclooxygenase 1 (COX1) signaling. Granule cargo release is required for full platelet responses induced by weak agonists or low concentrations of strong agonists. One of the important substances secreted from dense granules is ADP, which induces integrin activation and platelet aggregation through its receptors, P2Y_1_ and P2Y_12_ [1]. P2Y_1_ couples to Gα_q_ (G_q_) that transmits cellular signals mainly through its interaction and stimulation of phospholipase Cβ (PLCβ**)** [2]. Activation of PLCβ results in generation of inositol trisphosphate (IP3) and diacyl glycerol (DAG) that elicit calcium release and protein kinase C (PKC) activation, respectively [3]. P2Y_12_ couples to Gα_i_ (G_i_) family members [4], [5], primarily by coupling to G_i2_ [6], [7]. Activation of G_i_ downstream from P2Y_12_ inhibits cAMP accumulation and induces activation of the small GTPase Rap1b in a phosphoinositide 3-kinase (PI3K)-dependent manner.

Mice lacking P2Y_12_ exhibit highly prolonged bleeding times and impaired thrombus formation [5], [8]. P2Y_12_ deficient patients have increased bleeding times and abnormal platelet aggregation and secretion, probably due to abnormal cAMP regulation [9], [10]. Antiplatelet therapies targeting P2Y_12_, such as clopidogrel, ticagrelor, and prasugrel, have proven benefit in preventing or treating acute arterial thrombosis, and novel P2Y_12_ inhibitors are still in development [11], [12], [13], [14]. P2Y_12_ is required for ADP-induced platelet activation, and also contributes to platelet activation induced by other platelet agonists, such as thrombin and thromboxane A_2_ (TXA_2_) [8], [15]. Evidence supporting this notion comes not only from patients and mice lacking functional P2Y_12_, but also from the use of the adenosine-based P2Y_12_ antagonists, 2MeSAMP and Cangrelor [8], [10], [16], [17], [18]. However, a recent study reported that both 2MeSAMP and Cangrelor significantly increase intra-platelet cAMP levels in a P2Y_12_/G_i_-independent manner [19]. Thus, these findings raise a question not only about the specificity of these P2Y_12_ antagonists, but also about the role of P2Y_12_ in platelet activation.

Our data from studies using human platelets and P2Y_12_ deficient mice demonstrate that both 2MeSAMP and Cangrelor do not significantly raise cAMP levels, nor induce vasodilator-stimulated phosphoprotein (VASP) phosphorylation in platelets. Furthermore, 2MeSAMP and Cangrelor inhibit platelet aggregation and *in vivo* thrombosis only in wild-type mice, but not in P2Y_12_ deficient mice. Taken together, the results therefore indicate that these adenosine-based P2Y_12_ antagonists inhibit platelet function through the P2Y_12_-dependent mechanism.

## Materials and Methods

### Materials

α-Thrombin was purchased from Enzyme Research Laboratories (South Bend, IN). PAR 4 peptide AYPGKF was custom-synthesized at Biomatik USA, LLC (Wilmington, DE). FeCl_3_, ADP, and 2MeSAMP were from Sigma. Luciferase/luciferin reagent was from Chrono-log (Havertown, PA). Forskolin was purchased from Calbiochem (San Diego, CA). Fura-2/AM and Pluronic F-127 were from Invitrogen (Carlsbad, CA, USA). RalGDS-RBD fused to GST was a generous gift from Dr. Johannes L. Bos, University Medical Center, Utrecht, the Netherlands. Mouse monoclonal antibodies against VASP phosphorylated at residues serine 157 or serine 239 were purchased from Santa Cruz Biotechnology Inc. Rabbit monoclonal antibodies against the phosphorylated Ser^473^ or Thr^308^ residues of Akt were from Cell Signaling Technology (Beverly, MA). cAMP ELISA kit was from Amersham Biosciences.

### Animals

P2Y_12_ deficient mice were generated as described previously [5]. Littermate wild-type mice from heterozygous breeding were used as controls. All animal procedures were conducted in accordance with appropriate regulatory standards approved by the animal research committee at University of Kentucky, following institutional guidelines for the proper and humane use of animals in research.

### Preparation of Platelets

Washed mouse platelets were prepared as described previously [8]. Platelets were resuspended in modified Tyrode’s buffer (12 mM NaHCO_3_, 138 mM NaCl, 5.5 mM glucose, 2.9 mM KCl, 2 mM MgCl_2_, 0.42 mM NaH_2_PO_4_, 10 mM HEPES, pH 7.4) at 3×10^8^/ml, and incubated for 1 h at 22°C before use. Washed human platelets were prepared as described previously [8], and resuspended in modified Tyrode’s buffer. All participants were provided with written informed consent, and the study was approved by the research ethics boards at University of Kentucky.

### Platelet Aggregation and Secretion

Platelet aggregation at 37°C was measured by detecting changes in light transmission using a turbidometric platelet aggregometer (Chrono-Log) with stirring (1000 rpm). Platelet secretion was determined by measuring the release of ATP using luciferin/luciferase reagent. Luciferin/luciferase reagent (12 µl) was added to 238 µl of a washed platelet suspension 1 min before stimulation.

### Western Blot Analysis of Akt and VASP Phosphorylation in Platelets

Washed platelets (3×10^8^/ml) were preincubated with Cangrelor (1 µM), 2MeSAMP (10 µM), or forskolin (10 µM) for 5 min, and then stimulated with thrombin or AYPGKF in a platelet aggregometer at 37°C for 5 min and then solubilized in SDS-PAGE sample buffer. Platelet lysates were analyzed by SDS-PAGE on 4–15% gradient gels and immunoblotted using rabbit monoclonal antibodies specific for the phosphorylated Akt residues Ser^473^ or Thr^308^ [20]. To detect VASP phosphorylation, washed platelets were incubated with Cangrelor, 2MeSAMP, or forskolin at 37°C for 5 min. VASP phosphorylation was analyzed by Western blot as described previously [8].

### Determination of Intracellular cAMP Levels

Washed platelets (3×10^8^/ml) from healthy donors, P2Y_12_ deficient or wild-type mice were resuspended in Tyrode’s solution and incubated with 2MeSAMP, Cangrelor, or forskolin for 5 min at 37°C. The reaction was stopped by addition of an equal volume of ice-cold 12% (wt/vol) trichloroacetic acid. Samples were mixed and centrifuged at 2000 *g* for 15 minutes at 4°C. Each supernatant fraction was washed with 5 volumes of water-saturated diethyl ether 4 times and then lyophilized. cAMP levels were measured using a cAMP enzyme immunoassay kit [8].

To measure intra-platelet cAMP concentrations by mass spectrometry, cAMP was extracted from platelets (3×10^8^) by using a mixture of 3∶2 ice-cold acetonitrile and water. 2′ Deoxyadenosine 3′, 5′-cyclic monophosphate (2-dAcAMP) from Sigma Aldrich was used as an internal standard. The supernatant fraction containing cAMP was evaporated to dryness under N2 after centrifugation at 20,000 g for 30 min at 4°C, and reconstituted with 1∶1 acetonitrile and water. cAMP was quantitated by HPLC- electrospray ionization (ESI) tandem mass spectrometry using an AB Sciex (Foster City, CA) 4000 Q-Trap hybrid linear ion trap triple-quadrupole mass spectrometer equipped with a Turbo V electrospray ion source. cAMP and 2-dAcAMP were analyzed on a Kinetex PFP, 100×4.6 mm, 2.6 u column from Phenomenex with 0.1% formic acid in water and 0.1% formic acid in acetonitrile as solvents at a flow rate of 0.5 mL/min. The mass spectrometer was operated in the positive ESI mode monitoring the following MRM transitions: 314.13/135.9 and 314.13/118.9 for 2-dAcAMP; 330.21/136 and 330.21/118.9 for cAMP. Recovery was calculated using the internal standard and cAMP levels were determined using an off line calibration and normalized to platelet counts [21].

### Rap1b Activation

Washed platelets (3×10^8^/ml) from P2Y_12_ deficient mice were pre-incubated with 2MeSAMP (10 µM), Cangrelor (1 µM), or forskolin (10 µM) for 5 min, and then stimulated with thrombin (0.25 U/ml) or AYPGKF (500 µM) for an additional 5 min at 37°C with stirring. Rap1b activation was measured as described previously [22].

### Ca^2+^ Mobilization

Intra-platelet Ca^2+^ was measured using Fura-2/AM as described previously [22]. Labeled platelets were preincubated with Cangrelor (1 µM), 2MeSAMP (10 µM), forskolin (10 µM), or PGI_2_ (1 µM).

### *In vivo* Thrombosis

An *in vivo* thrombosis model was performed as described previously [23]. 20% of FeCl_3_ was applied to a filter paper disc that was immediately placed on top of the artery for 3 minutes. Cangrelor (4 µg per mouse) or saline was injected into the fundus oculi of the mice 5 min prior to the initiation of carotid artery injury.

### Statistics

Statistical significance was determined using a Student *t* test. Bar charts show mean ± standard deviation (SD). Fisher exact test was used for analysis of the *in vivo* thrombosis model. A *p*-value of less than 0.05 was considered significant.

## Results

### 2MeSAMP and Cangrelor Inhibit Platelet Activation through P2Y_12_

**Figure 1:**
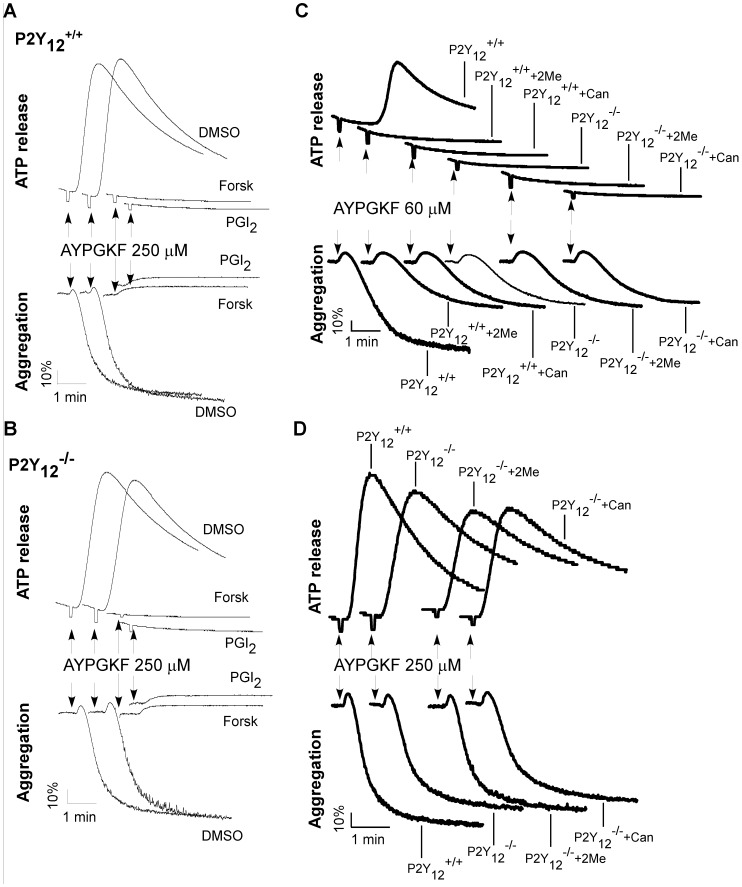
2MeSAMP and Cangrelor inhibit platelet aggregation and secretion through P2Y_12_-dependent mechanism. (A-B) Washed platelets from wild-type mice (A) or P2Y_12_ deficient mice (B) were pre-incubated with PGI_2_ (1 µM) or forskolin (10 µM) at 37°C for 5 min, and added with AYPGKF 250 µM to induce ATP release and aggregation. (C–D) Washed platelets (3×10^8^/ml) from P2Y_12_ deficient mice and littermate wild-type controls were pre-incubated with Cangrelor (1 µM) (Can) or 2MeSAMP (10 µM) (2Me) at 37°C for 5 min, and added with AYPGKF 60 µM (C) or 250 µM (D) to induce ATP release and aggregation. Data shown are representative of three independent experiments.

In order to determine whether inhibition of platelet activation by 2MeSAMP and Cangrelor is mediated specifically through P2Y_12_, the effects of 2MeSAMP and Cangrelor on platelet aggregation and secretion of ATP were examined. cAMP-dependent protein kinase (PKA) is a strong inhibitory signaling pathway of platelet activation. As expected, the PKA pathway activators, forskolin and PGI_2_, respectively inhibited aggregation and ATP secretion in both wild-type (Fig. 1A) and P2Y_12_ deficient platelets (Fig. 1B) in response to the thrombin receptor PAR4 activator, the peptide AYPGKF. In contrast, 2MeSAMP and Cangrelor inhibited aggregation and ATP release in wild-type platelets elicited only by low concentrations of AYPGKF (Fig. 1C). Similar to 2MeSAMP and Cangrelor treatment, aggregation and secretion were reduced in P2Y_12_ deficient platelets in response to low-dose AYPGKF (Fig. 1C), but not to high-dose AYPGKF (Fig. 1D). Unlike forskolin and PGI_2_, 2MeSAMP and Cangrelor did not affect aggregation and secretion of P2Y_12_ deficient platelets (Fig. 1C and 1D). These results not only demonstrate a role of the G_i_ pathway activated by ADP through P2Y_12_ in platelet activation in response to low-dose AYPGKF, but also indicate that the effects of 2MeSAMP and Cangrelor on platelet aggregation and secretion are P2Y_12_-dependent. ADP failed to stimulate aggregation and secretion of P2Y_12_ deficient platelets and 2MeSAMP- or Cangrelor-treated platelets (data not shown).

### 2MeSAMP and Cangrelor Failed to Stimulate cAMP Production in Platelets

**Figure 2:**
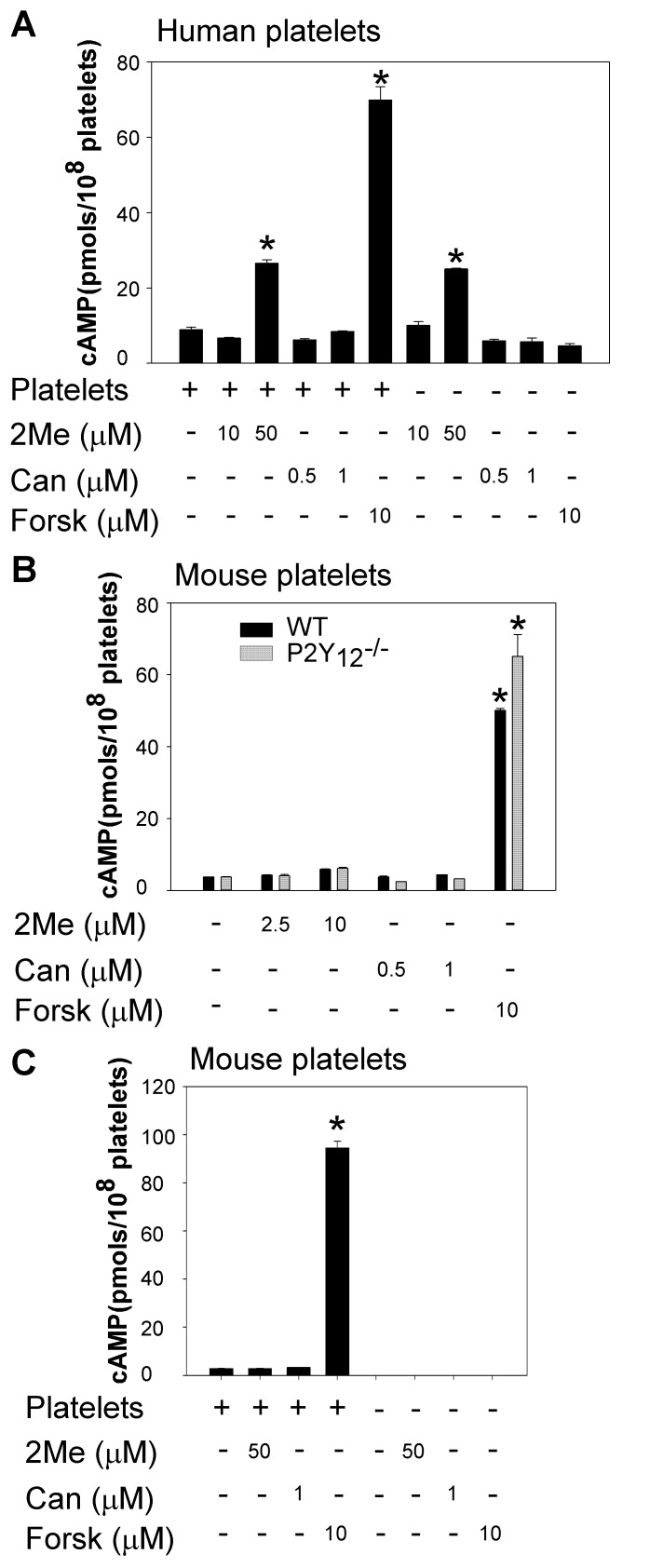
2MeSAMP and Cangrelor failed to stimulate cAMP production. (A) Washed human platelets were incubated with 2MeSAMP (2Me), Cangrelor (Can), or forskolin (Forsk) at 37°C for 5 min. The reactions were stopped by the addition of an equal volume of 12% (w/v) trichloroacetic acid. cAMP concentrations were determined by using a cAMP immunoassay kit. Statistical differences were examined by Student *t* test. Data are mean ± SD. *P<0.005 versus platelets in the absence of antagonists and forskolin. (B) Washed platelets from P2Y_12_ deficient mice or wild-type controls were pre-incubated with 2MeSAMP, Cangrelor, or forskolin for 5 min. The reactions were stopped by the addition of an equal volume of 12% (w/v) trichloroacetic acid. cAMP concentrations were determined by using a cAMP immunoassay kit. Statistical differences were examined by Student *t* test. Data are mean ± SD. *P<0.001 versus platelets of same genotype in the absence of antagonists and forskolin. (C) Washed platelets from wild-type mice were pre-incubated with 2MeSAMP, Cangrelor, or forskolin for 5 min. cAMP concentrations were determined by HPLC electrospray ionization tandem mass spectrometry as described under *Experimental Procedures*. Statistical differences were examined by Student *t* test. Data are mean ± SD. *P<0.001 versus platelets in the absence of antagonists and forskolin.

In contrast to a previous report showing that 2MeSAMP and Cangrelor increase intra-platelet cAMP concentrations through a P2Y_12_-independent mechanism [19], we found that Cangrelor, up to 1 µM, failed to increase intracellular cAMP levels in human or mouse platelets (Fig. 2A and 2B). As a positive control, forskolin markedly increased intra-platelet cAMP concentrations (Fig. 2A and 2B). While 2MeSAMP at concentrations at or below 10 µM did not increase cAMP levels in human platelets, it apparently increased intra-platelet cAMP levels at a higher concentration (50 µM) (Fig. 2A). Surprisingly, 2MeSAMP at 50 µM ‘enhanced’ cAMP concentrations in Tyrode’s solution in the absence of platelets. These results suggest that 2MeSAMP may cross-react with the cAMP ELISA assay, thereby causing false positive results. 2MeSAMP at concentrations at or below 10 µM did not increase cAMP levels in platelets from wild-type or P2Y_12_ deficient mice (Fig. 2B). Due to the fact that 2MeSAMP at high concentrations (>50 µM) interferes with the cAMP ELISA assay, we did not test high concentrations of 2MeSAMP in mouse platelets.

To further determine whether 2MeSAMP and Cangrelor can stimulate cAMP production in platelets, we developed an HPLC electrospray ionization tandem mass spectrometry method to measure cAMP concentrations. 2MeSAMP (50 µM) or Cangrelor (1 µM) failed to enhance cAMP levels in mouse platelets or Tyrode’s solution, as measured by this mass spectrometry-based assay (Fig. 2C). Using this assay we observed that forskolin stimulated cAMP production in platelets but not in solution in the absence of platelets.

### 2MeSAMP and Cangrelor Failed to Stimulate VASP Phosphorylation

**Figure 3:**
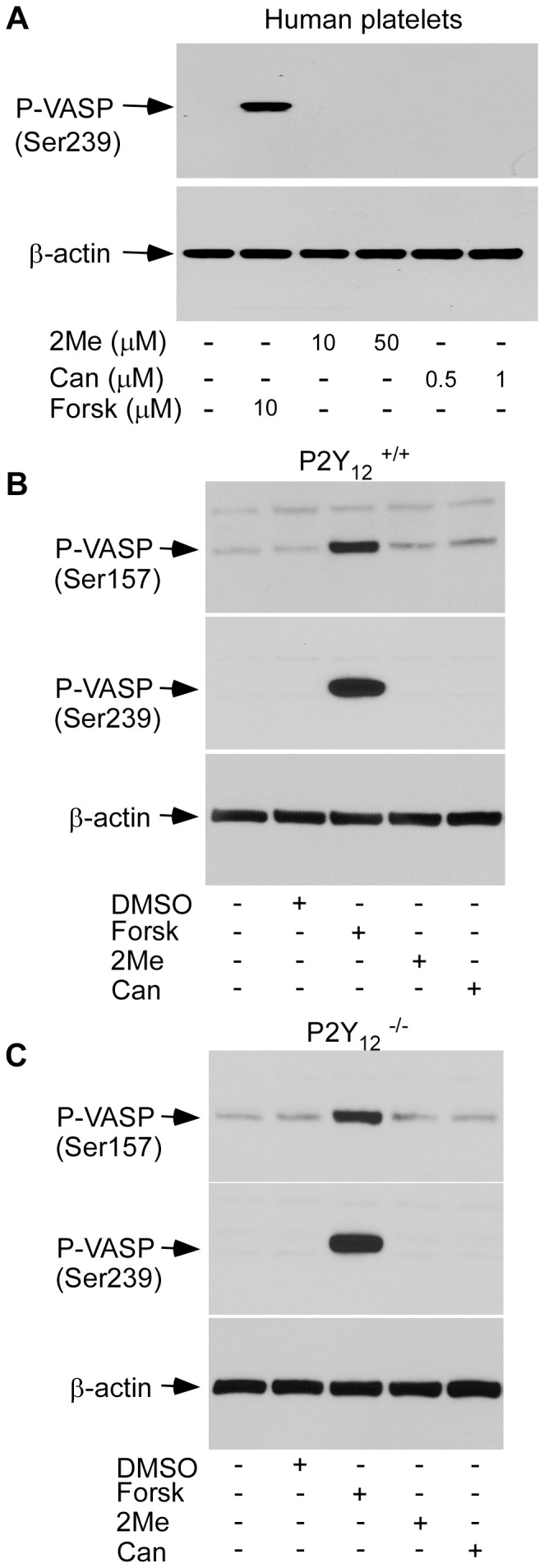
2MeSAMP and Cangrelor failed to stimulate VASP phosphorylation in platelets. (A) Washed human platelets were incubated with 2MeSAMP (2Me), Cangrelor (Can), or forskolin (Forsk) at 37°C for 5 min. The reactions were stopped by adding equal volume of 2×SDS sample buffer. Phosphorylation of VASP was detected by Western blotting with mouse monoclonal antibodies specifically recognizing the phosphorylated VASP residues Ser^239^. (B–C) Washed platelets from wild-type (B) or P2Y_12_ deficient mice (C) were pre-incubated with DMSO, forskolin (10 µM), 2MeSAMP (10 µM), or Cangrelor (1 µM) for 5 min. Reactions were stopped by adding equal volume of 2×SDS sample buffer. Phosphorylation of VASP was detected by Western blotting with mouse monoclonal antibodies specifically recognizing the phosphorylated VASP residues Ser^157^ or Ser^239^.

VASP is a well-known substrate for PKA [8], [24], [25]. Stimulation of platelets with forskolin induced VASP phosphorylation in human platelets (Fig. 3A). Thus, if 2MeSAMP and Cangrelor increase intracellular cAMP levels in platelets, they should also be able to induce VASP phosphorylation. However, neither 2MeSAMP nor Cangrelor, even at high concentrations, induced VASP phosphorylation in human platelets (Fig. 3A). 2MeSAMP and Cangrelor, respectively failed to induce VASP phosphorylation in both wild-type and P2Y_12_ deficient mouse platelets (Fig. 3B and 3C). These results indicate that 2MeSAMP and Cangrelor are unable to activate the cAMP pathway through a P2Y_12_-independent mechanism in platelets.

### Forskolin and PGI_2_, but not 2MeSAMP and Cangrelor, Inhibited AYPGKR-elicited Ca^2+^ Mobilization

**Figure 4:**
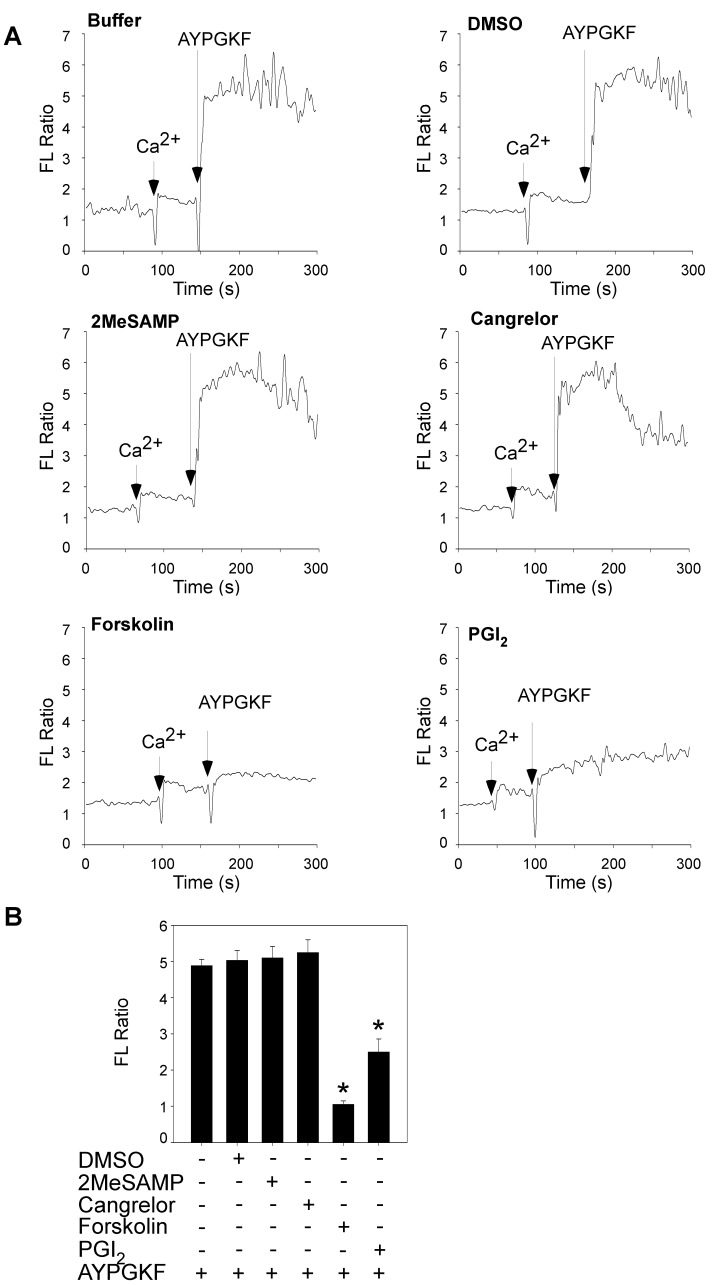
The PKA activators, but not 2MeSAMP and Cangrelor, inhibited Ca^2+^ mobilization in P2Y_12_ deficient platelets. Washed platelets from P2Y_12_ deficient mice were labeled with 12.5 µM Fura-2/AM/0.2% Pluronic F-127 and resuspended in Tyrode’s solution at 3×10^8^/ml. Platelets were preincubated with Cangrelor (1 µM), 2MeSAMP (10 µM), forskolin (10 µM), or PGI_2_ (1 µM), and then stimulated with AYPGKF (500 µM). Changes in the intracellular Ca^2+^ levels were measured every 2 s and expressed as a ratio of fluorescence (FL) detected at 509 nm emission with an excitation wavelength of 340 nm and 380 nm (A). Summarized data from three experiments are shown (B). Statistical differences were examined by Student *t* test. Data are mean ± SD. *P<0.001 versus DMSO.

PGE_1_, by activating G_s_-coupled receptors and increasing the generation of the intracellular cAMP, inhibited platelet activation in both wild-type and VASP deficient mice [26], demonstrating that cAMP/PKA signaling inhibits platelet activation through a VASP-independent mechanism. Stimulation of platelets with thrombin or TXA_2_ induces Ca^2+^ elevation through G_q_-dependent activation of PLC β. To determine whether or not PKA inhibition of platelet activation involves the PLC β/Ca^2+^ signaling, the effect of PGI_2_ and forskolin on AYPGKF-elicited Ca^2+^ elevation was examined. AYPGKF-induced Ca^2+^ mobilization was abolished by PGI_2_ and forskolin, respectively (Fig. 4). In contrast, 2MeSAMP and Cangrelor did not affect AYPGKF-elicited Ca^2+^ elevation in P2Y_12_ deficient platelets, supporting and extending our previous observation that, unlike PGI_2_ and forskolin, 2MeSAMP and Cangrelor cannot activate the cAMP pathway through a P2Y_12_-independent mechanism in platelets.

### Forskolin, but not 2MeSAMP and Cangrelor, Inhibited Akt Phosphorylation in P2Y_12_ Deficient Platelets

**Figure 5:**
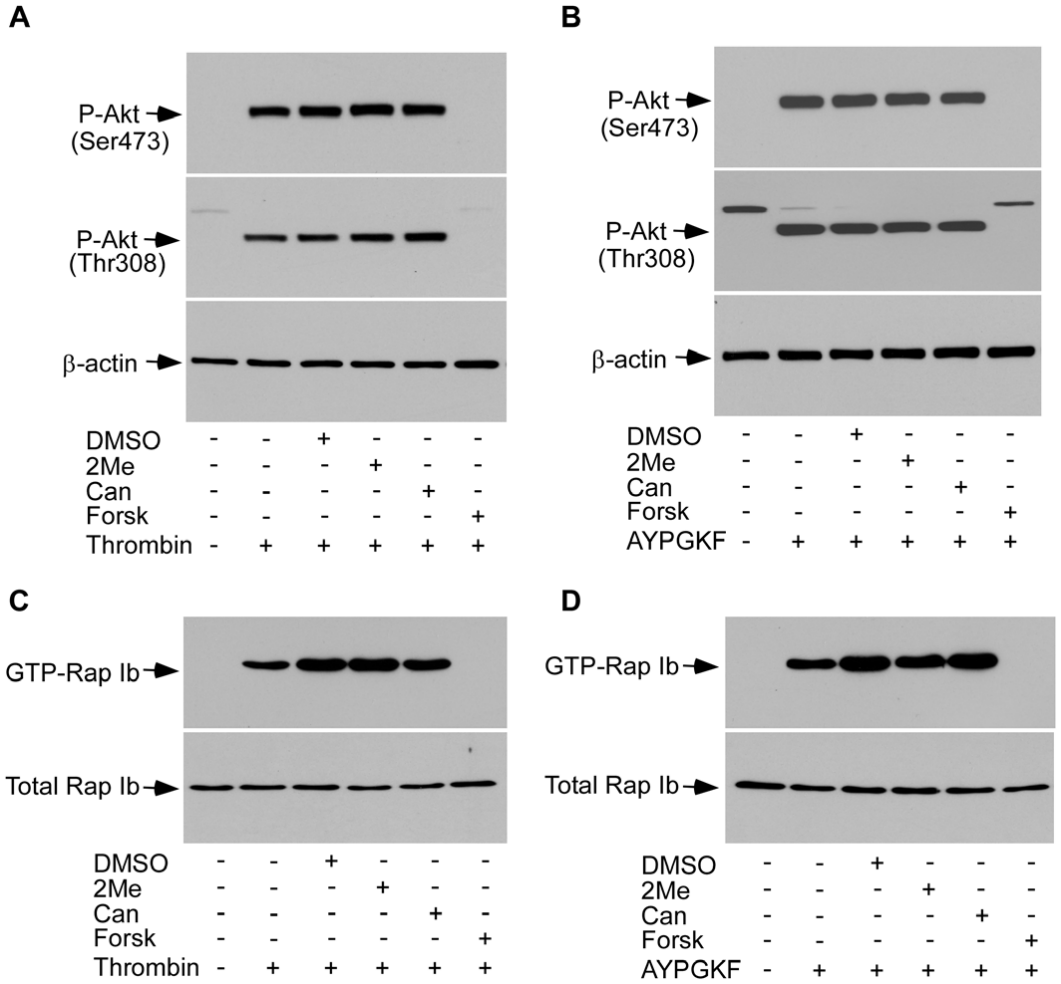
The PKA activators, but not 2MeSAMP and Cangrelor, inhibited Akt phosphorylation and Rap1b activation in P2Y_12_ deficient platelets. (A-B) Washed platelets from P2Y_12_ deficient mice were pre-incubated with 2MeSAMP (10 µM) (2Me), Cangrelor (1 µM) (Can), or forskolin (10 µM) (Forsk) at 37°C for 5 min, and then stimulated with thrombin (0.25 U/ml) (A) or AYPGKF (500 µM) (B). Akt phosphorylation was detected by Western blotting with a rabbit monoclonal antibody specifically recognizing the phosphorylated Akt residue Ser^473^ or Thr^308^. A mouse monoclonal antibody against β-actin (Sigma-Aldrich) was used to verify equal loading. (C–D) Washed platelets from P2Y_12_ deficient mice were pre-incubated with 2MeSAMP (10 µM) (2Me), Cangrelor (1 µM) (Can), or forskolin (10 µM) (Forsk) at 37°C for 5 min, and then stimulated with thrombin (0.25 U/ml) (C) or AYPGKF (500 µM) (D). GTP-bound Rap1 was precipitated with GST-RalGDS RBD bound to glutathione-agarose beads and detected by Western blot.

Akt phosphorylation in response to thrombin receptor stimulation involves both P2Y_12_-dependent and -independent mechanisms [20]. The effect of 2MeSAMP and Cangrelor on AYPGKF-induced Akt phosphorylation appears to be specifically mediated through the P2Y_12_-dependent pathway, because although 2MeSAMP and Cangrelor reduced AYPGKF-induced Akt phosphorylation in wild-type platelets (data not shown), they failed to inhibit Akt phosphorylation in P2Y_12_ deficient platelets in response to thrombin (Fig. 5A) or AYPGKF (Fig. 5B). In contrast, forskolin abolished Akt phosphorylation in P2Y_12_ deficient platelets elicited by thrombin (Fig. 5A) or AYPGKF (Fig. 5B).

### Forskolin, but not 2MeSAMP and Cangrelor, Inhibited AYPGKF-elicited Rap1b Activation in P2Y_12_ Deficient Platelets

Rap1b plays important roles in integrin inside-out signaling, and platelet aggregation [22], [27]. P2Y_12_/G_i_ signaling is critical for Rap1b activation in response to ADP and other agonists [28], [29], [30], [31]. However, we have recently shown that the thrombin mimetic PAR4 peptide AYPGKF and collagen at high concentrations elicited Rap1b activation in P2Y_12_ deficient platelets [22]. AYPGKF-induced, P2Y_12_-independent, activation of Rap1b requires Ca^2+^. Thus, if forskolin inhibits AYPGKF-induced Ca^2+^ elevation, it should also inhibit agonist-induced Rap1b activation in the P2Y_12_ deficient platelets. Indeed, forskolin inhibited thrombin- or AYPGKF-induced Rap1b activation in P2Y_12_ deficient platelets (Fig. 5C and 5D). In contrast, 2MeSAMP and Cangrelor did not affect thrombin- or AYPGKF-induced Rap1b activation in P2Y_12_ deficient platelets (Fig. 5C and 5D). These data demonstrate that 2MeSAMP and Cangrelor inhibited agonist-induced Rap1b activation in a P2Y_12_-specific manner.

### Cangrelor Inhibited Thrombus Formation in Wild-type but not P2Y_12_ Deficient Mice

**Figure 6:**
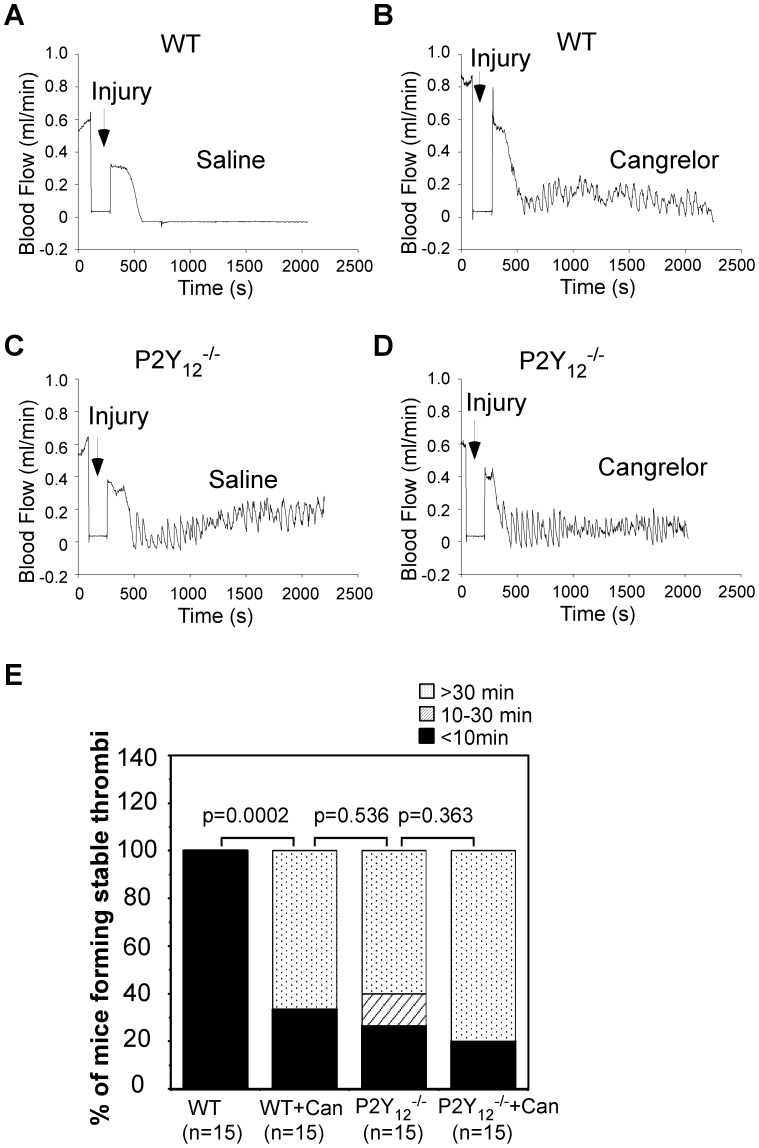
Cangrelor inhibited thrombus formation in wild-type but not P2Y_12_ deficient mice. (A–D) FeCl_3_-induced carotid artery injury was performed and time to occlusive thrombus formation recorded as described under *Experimental Procedures*. Wild-type (A and B) or P2Y_12_ deficient mice (C and D) were injected with saline (A and C) or Cangrelor (B and D) 5 min prior to the carotid artery injury. (E) Mice were calculated by time length of forming occlusive thrombi (<10 min, 10–30 min, >30 min). Statistical differences were examined by Fisher exact test.

All the above data indicate that the *in vitro* effects of 2MeSAMP and Cangrelor on platelets are mediated specifically through P2Y_12_. Unlike clopidogrel (Plavix), which is a pro-drug, Cangrelor is an active drug that does not require metabolic conversion for activity. Although the CHAMPION clinical trials did not show clinical benefit of cangrelor beyond that of clopidogrel for percuataneous coronary interventions [32], the BRIDGE study of short-term use of Cangrelor prior to surgery recently concluded with promising results [33]. Thus, it is important to demonstrate whether or not inhibition of thrombosis *in vivo* by Cangrelor is specifically mediated through P2Y_12_. The role of P2Y_12_ in *in vivo* thrombus formation was evaluated using a FeCl_3_-induced carotid artery thrombosis model. No occlusive thrombi formed in P2Y_12_ deficient mice when low-concentrations of FeCl_3_ (≤10%) were applied (data not shown). However, when 20% FeCl_3_ was used to cause vessel injury, P2Y_12_ deficient mice were able to initiate thrombus formation (Fig. 6C). Unlike wild-type mice, in which occlusive thrombi were formed within 10 minutes (Fig. 6A and 6E), thrombi were repeatedly formed and washed away in 60% (9 mice out of 15) of the P2Y_12_ deficient mice examined (Fig. 6C and 6E). Injection of Cangrelor into wild-type mice inhibited stable thrombus formation in a manner similar to that observed in P2Y_12_ deficient mice (Fig. 6B). In contrast, injection of Cangrelor failed to affect stable thrombus formation in P2Y_12_ deficient mice (Fig. 6D and 6E). These results demonstrate that Cangrelor mediated inhibition of thrombus formation *in vivo* is P2Y_12_ dependent.

## Discussion

The purpose of this study was to re-examine the specificity of the P2Y_12_ antagonists, Cangrelor and 2MeSAMP in inhibiting platelet functions. This study was initiated to test the assertion that Cangrelor and 2MeSAMP do not inhibit platelet activation solely through a P2Y_12_-dependent manner. Additionally, we discovered that inhibition of platelet activation by the cAMP/PKA pathway involves multiple signaling pathways including PLCβ, Akt, and Rap1b.

Cangrelor and 2MeSAMP have been extensively employed to identify the role of P2Y_12_ in platelet activation. However, a recent study reported that both Cangrelor and 2MeSAMP inhibit platelet activation by increasing intra-platelet cAMP concentrations through a P2Y_12_-independent mechanism [19]. If their data are correct, any conclusions on the role of P2Y_12_ in platelet activation drawn from experiments using these two antagonists need to be re-evaluated. More importantly, Cangrelor is being evaluated in clinical trials of thrombotic diseases with promising results [33]. Therefore, it is necessary to understand the detail mechanisms by which Cangrelor inhibits platelet activation. The key evidence showing that Cangrelor and 2MeSAMP inhibit platelet activation via a P2Y_12_-independent mechanism is that Cangrelor and 2MeSAMP dose-dependently increased intra-platelet cAMP levels in the absence of agonists. In that study, incubation of platelets with 0.05 µM of Cangrelor or 5 µM of 2MeSAMP dramatically increased cAMP concentrations. In contrast, our data indicate that Cangrelor (≤1 µM) and 2MeSAMP (≤10 µM) failed to stimulate cAMP production in both human and mouse platelets. We show that 1 µM of Cangrelor and 10 µM of 2MeSAMP are sufficient to inhibit P2Y_12_ function in isolated platelets (Fig. 1), which are the levels of those antagonists used to demonstrate the role of P2Y_12_ in most platelet studies [8], [18], [29], [30], [34], [35], [36]. The reason for the discrepancy between our study and the previous report are unknown. However, we found that 2MeSAMP at high concentrations (≥50 µM) apparently increases cAMP concentrations in platelets. But because these effects were observed when this compound was added to platelet-free Tyrode’s solution, we suspect that at high concentrations 2MeSAMP may cross-react with the antibody used for measurement of cAMP by ELISA and thereby produce a false positive result. To address this issue, we developed tandem mass spectrometry method to measure cAMP concentrations. In this highly specific and sensitive assay, neither 2MeSAMP (50 µM) nor Cangrelor (1 µM) enhanced cAMP concentrations in platelets or in solution. One difference we noticed between the previous study [19] and this study in measuring intra-platelet cAMP is that platelet-rich plasma was used in the previous study but we used washed platelets. It is not clear whether plasma affects cAMP measurement. Agents that elevate cAMP are strong inhibitors of platelet activation [37]. In agreement with the results that Cangrelor and 2MeSAMP could not induce cAMP production in platelets, they failed to inhibit platelet secretion and aggregation in P2Y_12_ deficient platelets. In contrast, PGI_2_ and forskolin, respectively abolished platelet activation in both wild-type and P2Y_12_ deficient mice.

Further evidence supporting the conclusion that Cangrelor and 2MeSAMP are unable to induce P2Y_12_-independent activation of the cAMP-PKA pathway in platelets was provided by our study of VASP phosphorylation. VASP is a well-established substrate for PKA. Thus, if Cangrelor and 2MeSAMP increase intra-platelet cAMP concentrations to a level similar as PGI_2_, they should be able to induce VASP phosphorylation. However, unlike PGI_2_ or forskolin, both Cangrelor and 2MeSAMP, even at high concentrations, failed to stimulate VASP phosphorylation in intact platelets.

Although it is well established that cAMP/PKA signaling inhibits platelet activation, the mechanisms by which cAMP/PKA inhibits platelet activation are not fully understood. In this regard, PKA can phosphorylate glycoprotein (GP) Ibβ, resulting in inhibition of GPIb-IX-V-stimulated platelet activation [38]. PKA has been shown to phosphorylate PLCβ and inhibit PLCβ signaling in COS 7 cells [39]. Ca^2+^ mobilization from the PLCβ signaling is a key event in platelet activation induced by GPCR agonists such thrombin and TXA_2_. Our data indicate that PKA-dependent inhibition of platelet activation involves the PLCβ pathway, because pre-treatment of platelets with forskolin or PGI_2_ abolished AYPGKF-elicited Ca^2+^ elevation (Fig. 4). Thus, if Cangrelor and 2MeSAMP inhibits platelet activation by increasing cAMP concentrations through P2Y_12_-independent mechanisms, they should be able to inhibit AYPGKF-elicited Ca^2+^ elevation. However, neither Cangrelor nor 2MeSAMP had an effect on AYPGKF-stimulated Ca^2+^ mobilization in P2Y_12_ deficient platelets.

Forskolin and PGI_2_, respectively inhibited multiple signaling pathways that are known to play important roles in mediating platelet secretion and aggregation, such as Akt phosphorylation [40] and Rap1b activation [27]. In contrast, Cangrelor and 2MeSAMP had no effect on AYPGKF-stimulated phosphorylation of Akt and Rap1b activation in P2Y_12_ deficient platelets. These data demonstrate that the inhibitory effect of Cangrelor and 2MeSAMP on platelet activation is P2Y_12_-dependent and does not involve the P2Y_12_-independent activation of the cAMP/PKA pathway.

P2Y_12_ is not only important for agonist-induced platelet activation *in vitro*, but also contributes to thrombosis *in vivo* [8], [41]. Using a FeCl_3_-induced carotid artery thrombosis model, we found that the initial thrombus formation is not impaired in P2Y_12_ deficient mice. However, most P2Y_12_ deficient mice cannot form stable thrombi under our experimental conditions. Likewise, injection of Cangrelor into wild-type mice inhibits stable thrombus formation. Accordingly, injection of Cangrelor has no effect on thrombus formation in P2Y_12_ deficient mice. Initial thrombus formation caused by vascular injury is mediated by von Willebrand factor bound to subendothelial matrix. Stable thrombus formation requires integrin activation, a process in which P2Y_12_ plays an important role. Our results demonstrate that Cangrelor inhibition of thrombus formation *in vivo* is also P2Y_12_ dependent.
